# Establishment of a Virus-Induced Gene-Silencing (VIGS) System in Tea Plant and Its Use in the Functional Analysis of *CsTCS1*

**DOI:** 10.3390/ijms24010392

**Published:** 2022-12-26

**Authors:** Guodong Li, Yan Li, Xinzhuan Yao, Litang Lu

**Affiliations:** 1 The Key Laboratory of Plant Resources Conservation and Germplasm Innovation in Mountainous Region (Ministry of Education), College of Life Sciences, Guizhou University, Guiyang 550025, China; 2 College of Tea Sciences, Institute of Plant Health & Medicine, Guizhou University, Guiyang 550025, China

**Keywords:** tea plant, virus-induced gene silencing (VIGS), tobacco rattle virus (TRV), *CsPOR1*, *CsTCS1*

## Abstract

Tea (*Camellia sinensis* [L.] O. Kuntze) is an important global economic crop and is considered to enhance health. However, the functions of many genes in tea plants are unknown. Virus-induced gene silencing (VIGS) mediated by tobacco rattle virus (TRV) is an effective tool for the analysis of gene functions, although this method has rarely been reported in tea plants. In this study, we established an effective VIGS-mediated gene knockout technology to understand the functional identification of large-scale genomic sequences in tea plants. The results showed that the VIGS system was verified by detecting the virus and using a real-time quantitative reverse transcription PCR (qRT-PCR) analysis. The reporter gene *CsPOR1* (protochlorophyllide oxidoreductase) was silenced using the vacuum infiltration method, and typical photobleaching and albino symptoms were observed in newly sprouted leaves at the whole plant level of tea after infection for 12 d and 25 d. After optimization, the VIGS system was successfully used to silence the tea plant *CsTCS1* (caffeine synthase) gene. The results showed that the relative caffeine content was reduced 6.26-fold compared with the control, and the level of expression of *CsPOR1* decreased by approximately 3.12-fold in plants in which *CsPOR1* was silenced. These results demonstrate that VIGS can be quickly and efficiently used to analyze the function of genes in tea plants. The successful establishment of VIGS could eliminate the need for tissue culture by providing an effective method to study gene function in tea plants and accelerate the process of functional genome research in tea.

## 1. Introduction

VIGS is an RNA-silencing technology that is based on the mechanism of plant resistance to viral infections [1,2] and is specifically induced by viral replication and transcription. Viral vectors with specific exogenous genes that contain double-stranded RNA (dsRNA) play an important role in the process of VIGS. The dsRNAs are degraded into small interfering RNA (siRNAs) that vary in length from 21 to 23 nt and are processed by a Dicer-like nuclease. These siRNAs target the corresponding plant gene mRNAs, causing their degradation [3]. The mRNA of homologous gene sequences is degraded or modified by methylation, which causes changes in the plant phenotype or physiological indicators, thus, enabling the identification of functional genes [4,5]. VIGS technology does not require genetic transformation, and it can be easily used to acquire mutants. With its advantages of high speed, low cost, and high-throughput, VIGS technology currently serves as one of the most attractive technological tools in the field of functional genomics research.

Kumaga et al. [1] first applied VIGS technology to plants and inserted the tobacco *PDS* (phytoene desaturase) gene into tobacco mosaic virus (TMV). Inoculation with the altered TMV resulted in epistatic leaves that were chlorotic and whitened, which was accompanied by a significant decrease in the level of *PDS* mRNA. However, the TMV-induced virus had a short duration of silent phenotype, which limited its application. Tobacco rattle virus (TRV) is advantageous for its wide host range, high silencing efficiency, and long duration; thus, it has been widely used in a variety of plants [6,7], including strawberry (*Fragaria × ananassa* Duch.) [8], cotton (*Gossypium* spp.) [9], and potato (*Solanum tuberosum* L.) [10]. In addition, some economic trees have also been successfully established based on the system, including lychee (*Litchi chinensis* Sonn.) [11]. TRV has become the most widely used virus in the construction of VIGS systems.

The enzyme protochlorophyllide oxidoreductase (*POR*) catalyzes a light-dependent step in chlorophyll biosynthesis that is essential to photosynthesis, which catalyzes the reduction in the photosensitizer and substrate protochlorophyllide to form the pigment chlorophyllide [12,13,14,15]. Based on the function of the *POR* gene, it was used as a visual gene in the VIGS systems of *Ananas bracteatus* var. *pineapple*, *Nicotiana benthamiana*, and *Arabidopsis thaliana*, and a photo-bleaching phenomenon occurred after *POR1* silencing [16,17].

Tea plants (*Camellia sinensis* [L.] O. Kuntze) are one of the most important economic trees in China and have been cultivated for thousands of years. Currently, with the development of tea genomics [18], an increasing number of perfect genomic libraries have been established (http://bigd.big.ac.cn/gsa/s/21CxCeKK, http://tpia.teaplant.org, accessed on 8 June 2022). However, there are relatively few methods to analyze the function of genes in tea plants, and the lack of studies on the stable genetic transformation of tea plants has caused a bottleneck in the research on tea biology. However, a TRV-VIGS system is highly efficient, takes less time, and is relatively simple to perform; therefore, it can compensate for the problems described above and enable the quick and accurate study of gene functions [19,20]. Caffeine is one of the most important components of tea plants, and it has a crucial impact on the quality of tea. *CsTCS1* (caffeine synthase) catalyzes the last two steps of caffeine synthesis; the level of its expression affects the content of caffeine in the tea plants [21]. Yu et al. [22] transformed *CsTCS1* into tobacco by heterologous transformation. The researchers detected the presence of caffeine synthase in tobacco, but the transformants did not produce caffeine. Mohanpuria et al. [23] used RNA interference (RNAi) technology to knock out *CsTCS1* in tea plants, which reduced the contents of caffeine and theobromine in the gene knockdown tea plant. Deng et al. introduced fragments of *CsTCS1* into *E. coli* to establish a prokaryotic expression system. The results showed that caffeine was present in its extracellular secretions. To our knowledge, there are currently no reports of the use of VIGS in tea plants. In this study, the TRV-mediated VIGS system of tea plants was established using the reporter gene *CsPOR1*, which encodes protochlorophyllide oxidoreductase. This system was used to silence *CsTCS1*. The results accurately determined the function of related endogenous genes in tea plants, which should improve the breeding efficiency of tea plants and create new varieties. Therefore, the establishment of the tea plant VIGS system can improve the efficiency of tea gene function research, avoid the problem of the incomplete or non-expression of tea plant endogenous genes in heterologous model organisms, introduce a new way to study the gene function of tea, and provide innovative tea plant germplasm. This study aimed to establish a TRV-mediated VIGS system for tea plants.

## 2. Results

### 2.1. TRV Can Infect Fuding Dabaicha

**Figure 1:**
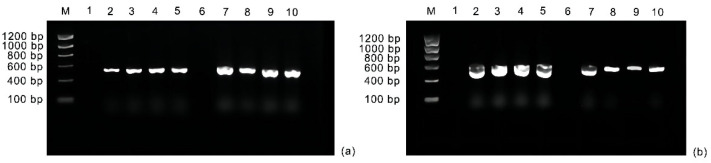
Detection of TRV transcripts in VIGS leaves with phenotypic changes of tea plants: (**a**). 1–5 detected the strand of viral pTRV2, and 6–10 detected the strand of viral pTRV1 (1, uninfected seedlings; 6, uninfected cuttings; 2, seedlings infected with pTRV1 + pTRV2; 7, cuttings infected with pTRV1 + pTRV2; 3–5, seedlings infected with pTRV1 + pTRV2-*CsPOR1*; 8–10, cuttings infected with pTRV1 + pTRV2-*CsPOR1*); (**b**) 1–5 detected the strand of viral pTRV1, and 6–10 detected the strand of viral pTRV2 (1, uninfected seedlings; 6, uninfected cuttings; 2, seedlings infected with pTRV1 + pTRV2; 7, cuttings infected with pTRV1 + pTRV2; 3–5, seedlings infected with pTRV1 + pTRV2-*CsTCS1*; 8–10, cuttings infected with pTRV1 + pTRV2-*CsTCS1*). M: DL1200 marker.

Three groups of plants were used to demonstrate that Fuding Dabaicha can be infected with tobacco rattle virus (TRV). The first group consisted of the newly sprouted leaves, including photobleached leaves, albino leaves, reddish leaves, and pale pink leaves of plants that were infected by *Agrobacterium* that harbored pTRV1 + pTRV2-*CsPOR1*, and *Agrobacterium* that harbored pTRV1 + pTRV2-*CsTCS1*. The second group consisted of the newly sprouted leaves that were not silenced and were treated with *Agrobacterium* that harbored the empty vectors pTRV1 and pTRV2. The final group consisted of untreated newly sprouted leaves that had not been treated with *Agrobacterium*. The leaves were randomly collected for RT-PCR detection. The results clearly showed that the transcripts of viral pTRV1 and pTRV2 were successfully detected in newly germinated tea leaves from plants that were infected with the virus (Figure 1). Simultaneously, we observed varying degrees of black spots in the newly reddish and albino leaves of the *CsPOR1*-silenced plants (Figure S1), which were attributed to the background TRV viral effect, whereas the black spot symptoms were not observed in the control group pTRV2. This also indicated that TRV had successfully infected the tea plants, and these phenomena are consistent with those described by Di Stilio et al. [24]. This shows that recombinant TRV can effectively replicate and spread in the Fuding Dabaicha plants.

As described by Kalbande and Patil [25], contamination with *Agrobacterium* was checked using the primers *oriV*-F-5′-CTACGGCCAGGCAATCTACC-3′ and *oriV*-R-5′-GAGCTGCATGTGTCAGAGGT-3′ (designed to amplify 130 bp of the *oriV* gene on the Ti plasmid). All eight putative transformants were tested using all the primers described above, which included the following: four-column-infected tea plants with pTRV1 + pTRV2, two tea plants infected with pTRV1 + pTRV2-*CsPOR1*, and two tea plants infected with pTRV1 + pTRV2-*CsTCS1*). The PCR with *oriV* primers confirmed the absence of *Agrobacterium* itself as a pathogen in eight putative transformants and wild-type plants (Figure S4).

### 2.2. TRV-Mediated VIGS System Was Successfully Used in the Tea plants

#### 2.2.1. The TRV-Mediated VIGS System Was Successfully Used in the Tea Seedlings

**Figure 2:**
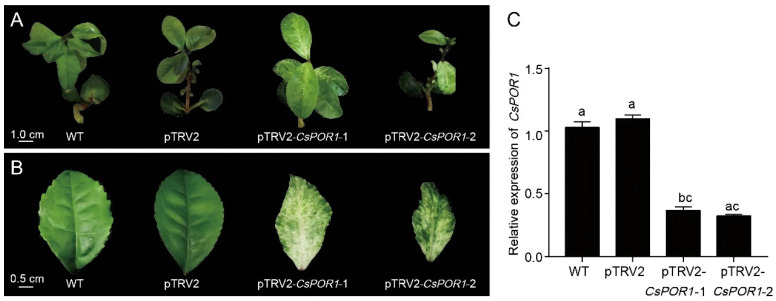
TRV-mediated VIGS of the *CsPOR1* gene in tea seedling leaves. (**A**,**B**) WT, wild-type tea seedlings; pTRV2, infected seedlings with pTRV1 + pTRV2 *Agrobacterium*; pTRV2-*CsPOR1*-1 and pTRV2-*CsPOR1*-2, infected seedlings with pTRV1 + pTRV2-*CsPOR1 Agrobacterium*. (**C**) The expression of *CsPOR1* was down-regulated in the photo-bleached leaves of tea seedlings. WT, wild-type tea seedlings; pTRV2, infected with pTRV1 + pTRV2 *Agrobacterium*; pTRV2-*CsPOR1*-1 and pTRV2-*CsPOR1*-2, infected with pTRV1 + pTRV2-*CsPOR1 Agrobacterium.* The error bars represent the mean ± SE of the three independent experiments. Different lowercase letters indicate a significant difference at *p* < 0.05 by Duncan’s multiple range test. SE, standard error; TRV, tobacco rattle virus; WT, wild type.

To test whether the TRV-based vector can effectively induce the silencing of endogenous genes in tea seedlings, *CsPOR1* was selected as the visual marker. *POR1* is one of the key genes in chlorophyll biosynthesis. Therefore, inhibition of this gene will lead to photodegradation of the chlorophyll or changes in the green color of plants. All the tea seedlings that were treated with the TRV virus survived, indicating that the vacuum-infiltration method can be used for VIGS experiments. All the newly developed leaves of the wild-type tea seedlings and those that were infected with pTRV1 + pTRV2 *Agrobacterium* were a normal shade of green after 25 d. Simultaneously, five of the tea seedlings infected with pTRV1 + pTRV2-*CsPOR1 Agrobacterium* exhibited a photo-bleached phenotype in the leaves that grew after 25 d (Figure 2A). Two of the tea seedlings with obvious photo-bleaching symptoms were selected as the materials for the qRT-PCR experiments, and they were then named pTRV2-*CsPOR1*-1 and pTRV2-*CsPOR1*-2. The symptoms of photo-bleaching in the leaves of the silenced plants became increasingly significant as the plants grew (Figure 2B). To further verify whether *CsPOR1* was silent, the level of *CsPOR1* mRNA in the leaves infected with pTRV2 + pTRV2-*CsPOR1* was measured using a qRT-PCR. Compared with the wild-type tea seedlings and those infected with pTRV1 + pTRV2, the content of the *CsPOR1* transcripts was significantly reduced in the leaves infected with pTRV1 + pTRV2-*CsPOR1* (Figure 2C). These observations indicated that the TRV-mediated VIGS system could silence the visual marker gene (*CsPOR1*) in the tea seedling leaves. The results also showed that the TRV-VIGS system was successfully applied to tea seedlings and could significantly reduce the level of expression of endogenous target genes in the leaves, thereby achieving gene silencing.

#### 2.2.2. TRV-Mediated VIGS System Was Successfully Used in the Tea Cuttings

**Figure 3:**
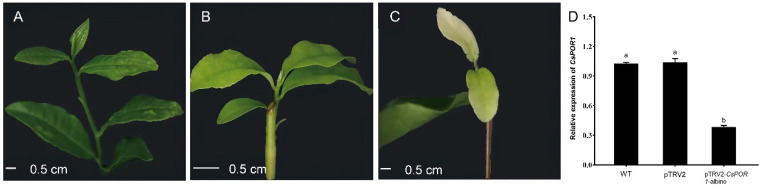
TRV-mediated VIGS of the *CsPOR1* gene in tea-cutting leaves. (**A**) Untreated tea cuttings. (**B**) New leaves with normal symptoms 20 d after infection with pTRV1 + pTRV2. (**C**) New leaves with albino symptoms 20 d after infection with pTRV1 + pTRV2-*CsPOR1*. (**D**) Expression of *CsPOR1* was downregulated in the leaves of tea cuttings that were albino or reddish. WT, uninfected cuttings; pTRV2, infection with pTRV1 + pTRV2 *Agrobacterium*; pTRV2-*CsPOR1*-albino and pTRV2-*CsPOR1*-reddish, infection with pTRV1 + pTRV2-*CsPOR1 Agrobacterium*. The error bars represent the mean ± SE of the three independent experiments. Different lowercase letters indicate significant differences at *p* < 0.05 by Duncan’s multiple range test. qRT-PCR, real-time quantitative reverse transcription PCR; SE, standard error; TRV, tobacco rattle virus; VIGS, virus-induced gene silencing.

The vacuum infiltration method, which was developed by increasing the amount of sonication, was used to infect the tea cuttings. New sprouting leaves of uninfected tea cuttings and those infected with pTRV1 + pTRV2 *Agrobacterium* were still green after 20 d of infiltration (Figure 3B,C). However, the leaves of tea cuttings infected with pTRV1 + pTRV2-*CsPOR1 Agrobacterium* that had grown for 20 d were albino (Figure 3A). Among the 23 tea cuttings, the new leaves of eight cuttings were albino. The efficiency of silencing was 34.7%. One of the silenced plants was selected as experimental material for the qRT-PCR. The cuttings of the albino leaves were named pTRV2-*CsPOR1*-albino. The qRT-PCR results revealed that the expression of *CsPOR1* in the leaves that were albino (Figure 3A) was successfully knocked down, as shown in Figure 3D. The results also indicated that the VIGS system can still be used successfully in tea cuttings.

### 2.3. Optimized VIGS System in Tea

Based on the TRV-VIGS system of the tea seedlings, a TRV-VIGS system suitable for the tea cuttings was explored using the osmotic buffer that that was used for it. The amount of MgCl_2_ in the buffer was increased to 16 g·L^−1^, and the duration of the contact between the plant and the mixed bacterial solution was extended from 1 h to 2 h. An ultrasonic treatment step was added before the vacuum infiltration. The OD_600_ and vacuum pressure values suitable for the TRV-VIGS of the tea seedling system were explored (Table 1 and Table 2). The results indicated that the most effective conditions to infiltrate the tea seedlings included the following: an OD_600_ of 1.2, vacuum pressure of 0.7 kPa, and osmosis buffer of 4.74 g·L^−1^ of MS, 2 mol·L^−1^ of 6-BA, 2 mol·L^−1^ of As, and 100 umol·L^−1^ of NAA. The silencing efficiency of this system was 12.5%. Most of the tea cuttings were successfully infiltrated at an OD_600_ of 1.2, vacuum pressure of 0.8 kPa, and osmosis buffer of 4.74 g·L^−1^ of MS, 2 mol·^−1^ of 6-BA, 2 mol·L^−1^ of As, 100 umol·L^−1^ of NAA, and 16 g·L^−1^ of MgCl_2_, and the silencing efficiency of this system was 34.7%.

### 2.4. Prediction of Gene Silencing Times by the Phenotypic Changes of CsPOR1-Silenced Plants

**Figure 4:**
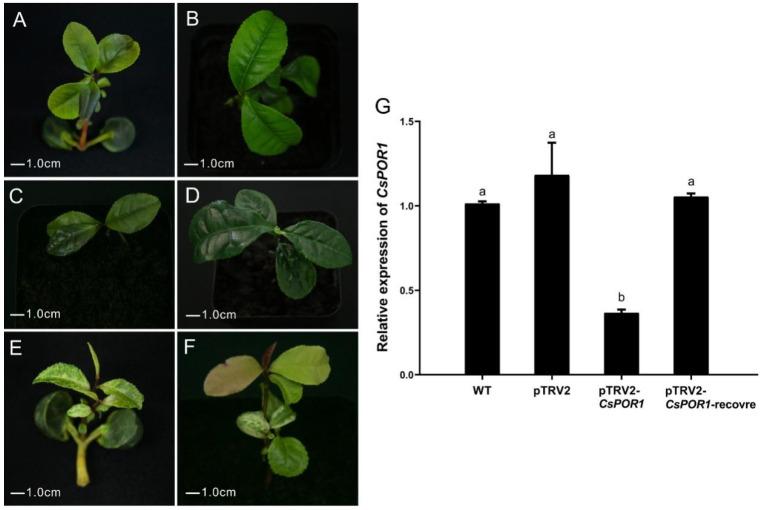
Leaf phenotypic and *CsPOR1* expression changed after treatment with pTRV1 + pTRV2-*CsPOR1.* (**A**,**B**) Phenotype of tea seedling leaves at 25 d and 65 d after infection with *Agrobacterium* that harbored pTRV1 + pTRV2. (**C**,**D**) Phenotype of the leaves at 25 d after infection and 65 d of the control plants that had not been treated. (**E**,**F**) Phenotype of the leaves at 25 d and 65 d after infection with pTRV1 + pTRV2-*CsPOR1 Agrobacterium*. (**G**) The relative level of changes in the expression of *CsPOR1* gene after the leaves had recovered phenotypically. WT, wild-type tea seedlings; pTRV2, tea seedlings infected with pTRV1 + pTRV2; pTRV2-*CsPOR1* and pTRV2-*CsPOR1*-recovered, tea seedlings infected with pTRV1 + pTRV2-*CsPOR1*. The error bars represent the mean ± SE of three independent experiments. Different lowercase letters indicate significant differences at *p* < 0.05 by Duncan’s multiple range test. SE, standard error; TRV, tobacco rattle virus; WT, wild type.

The tea seedlings were infected by pTRV1 + pTRV2-*CsPOR1 Agrobacterium*, and the nascent leaves that grew after 25 d in the infected part of the upper plant exhibited symptoms of photo bleaching (Figure 4E). Newly sprouted leaves of the wild-type tea seedlings and those infected with pTRV1 + pTRV2 *Agrobacterium* were still green at 25 d after infiltration (Figure 4A,C). After 65 d, the young leaves of those plants infected with pTRV1 + pTRV2-*CsPOR1 Agrobacterium* were restored to green (Figure 4F). However, after 65 d, the leaves on the wild-type plants and those infected with pTRV1 + pTRV2 *Agrobacterium* still remained green (Figure 4B,D). The photo-bleaching phenomenon was observed to last for more than 40 d.

Newly sprouted leaves after 65 d from the untreated plants (wild-type), non-silenced plants (vacuum-infiltrated with pTRV1 + pTRV2 *Agrobacterium*), those silenced for green leaves (vacuum-infiltrated with pTRV1 + pTRV2-*CsPOR1 Agrobacterium*), and the photo-bleached leaves after 25 d of silenced plants (vacuum-infiltrated with pTRV1 + pTRV2-*CsPOR1 Agrobacterium*) were collected for the qRT-PCR analysis. The silenced plants that exhibited photo-bleached leaves were named pTRV2-*CsPOR1*. The silenced plants with green leaves were named pTRV2-*CsPOR1*-recover. The qRT-PCR results confirmed that the expression of *CsPOR1* was still knocked down in the photo-bleached leaves of the infected plants and recovered when the leaves turned green (Figure 4G).

### 2.5. CsTCS1 Silencing Inhibited the Formation of Caffeine in the Leaves of Fuding Dabaicha

**Figure 5:**
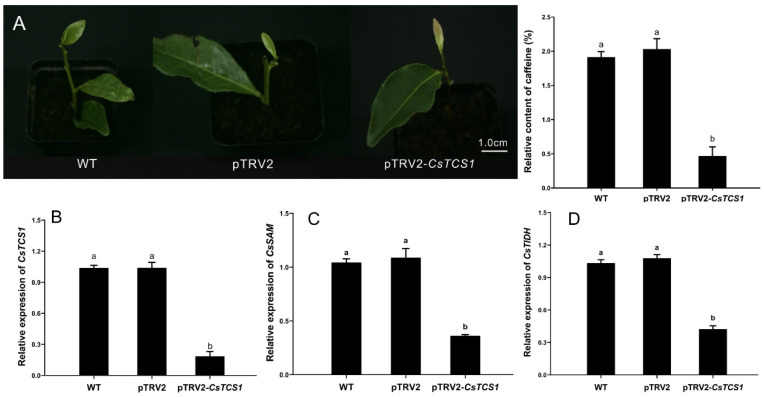
Silencing of *CsTCS1* in a leaf of tea plant using the VIGS system. (**A**) Relative content of caffeine. WT, untreated tea cuttings; pTRV2, tea cuttings infected by pTRV1 + pTRV2 *Agrobacterium*; pTRV2-*CsTCS1*, tea cuttings infected by pTRV1 + pTRV2-*CsTCS1 Agrobacterium*. (**B**–**D**) The relative level of expression of *CsTCS1* (**B**), *CsSAM* (**C**), *CsTIDH* (**D**). WT, untreated tea cuttings; pTRV2, vacuum-infiltrated with pTRV1 + pTRV2; pTRV2-*CsTCS1*, vacuum-infiltrated with pTRV1 + pTRV2-*CsTCS1*. The error bars represent the mean ± SE of the three independent experiments. Different lowercase letters indicate significant differences at *p* < 0.05 by Duncan’s multiple range tests. SE, standard error; TRV, tobacco rattle virus; VIGS, virus-induced gene signaling.

The level of expression of *CsTCS1* in the tea plant leaves was downregulated to study the function of *CsTCS1* using the TRV-VIGS system. In this study, tea cuttings were selected as experimental materials to measure the relative content of caffeine, in order to avoid interference in the experimental results caused by the individual differences of tea seedlings. The cuttings infected with pTRV1 + pTRV2-*CsTCS1* grew new leaves that were pale pink after 12 d. Four of the 20 treated cuttings exhibited these symptoms, and the cutting with the most significant pink symptoms was selected for the experiment and named pTRV2-*CsTCS1*. The silencing efficiency of *CsTCS1* was 20%. The newly developed leaves of the pTRV1 + pTRV2 cuttings treated with *Agrobacterium* and the wild-type cuttings were normal green. The content of caffeine in the new leaves of the cuttings was determined using HPLC (Figure S2). Compared with the green leaves of the wild-type cuttings and those infected with pTRV1 + pTRV2, the content of caffeine decreased by 6.26-fold in the pale pink leaves of cuttings infected with pTRV2-*CsTCS1* (Figure 5A). This is consistent with the results described by Mohanpuria et al. [23]. To detect the relative level of expression of *CsTCS1* and the changes in levels of expression of the key genes involved in caffeine synthesis after silencing, the levels of expression of *CsTCS1*, *CsSAM* (S-adenosylmethionine), and *CsTIDH* (hypoxanthine nucleotide dehydrogenase) were determined by a qRT-PCR. The results showed that the level of expression of *CsTCS1* was significantly reduced (Figure 5B). Simultaneously, the levels of expression of *CsSAM* and *CsTIDH* were also significantly reduced (Figure 5C,D).

## 3. Discussion

VIGS technology has been widely used in gene function analysis, particularly in plants that are difficult to transform genetically, such as *PeHSF (Populus euphratica)* [26], Chinese narcissus (*Narcissus tarzetta* L.) *NtMYB3* [27], *Jatropha curcas JcKASII* [28], and *Malus* crabapple *McMYB10* [29]. However, there have been no reports on the use of VIGS technology in tea plants. In this study, a VIGS transformation system for the tea plant was established, which lays the foundation for the analysis of gene functions in tea plants.

There are multiple reporter genes in plants, but they vary greatly given plant diversity. Whether the genes are anthocyanin-dominant or chlorophyll- or carotenoid-dominant is uncertain, and reporter genes should be selected based on the plant characteristics [29,30,31]. *PDS*, *CHS* (chalcone synthase), *Ftsh* (filamentation temperature sensitive H), *ChlH* (magnesium chelatase subunit H), and the *POR1* genes are commonly used as reporter genes in VIGS systems. Kumagai et al. [1] used *PDS* as a marker gene in *Nicotiana tabacum* L.; Chen et al. [30] used *CHS* as a marker gene in *Petunia hybrida*; Saitoh and Terauchi [32] used *Ftsh* as a reporter gene in *Nicotiana tabacum* L.; Jia et al. [33] used *ChlH* as a reporter gene in *Prunus persica*; and Li and Ma [17] used *POR1* as a marker gene in *Ananas comosus* var. *bracteatus*. This study used tea plant *CsPOR1* as a marker gene. We chose the 308 bp coding sequence of *CsPOR1* as a target gene fragment to insert into the TRV2 vector to construct pTRV2-*CsPOR1*, followed by successful silencing of the *POR1* gene. This result is consistent with a previous report that fragments of 200–1300 bp can be used to induce gene silencing [34]. The silencing of *CsPOR1* in the tea plant appeared to demonstrate a photo-bleaching phenomenon.

Previous studies have indicated that the vacuum infiltration methods are suitable for VIGS in younger seedlings and woody plants [35,36]; therefore, this study selected the vacuum infiltration technique as the best method of infection. The infiltration conditions, such as *Agrobacterium* solution and vacuum pressure, play important roles in the efficiency of VIGS [29]. In this study, we chose different combinations to explore the best OD_600_ and vacuum pressure. The results showed that an OD_600_ of 1.2 and an osmotic pressure of 0.7 kPa provided the best conditions for the infiltration of the tea plant sexual systems to utilize VIGS. The silencing efficiency was 12.5%. In addition, an OD_600_ of 1.2 and an osmotic pressure of 0.8 kPa provided the most effective infiltration conditions for the tea plant clones. In this case, the silencing efficiency was 34.7%. This system successfully transferred the TRV vector to tea plants. Some studies have suggested that cultivating plants at room temperature between 22 and 25 °C could help to increase the intensity of gene silencing by prolonging its duration [19,37]. Therefore, in this experiment, the temperature was maintained at 25 °C to obtain the best silencing efficiency, which is consistent with the previous data described above.

The plant material affects the efficiency of TRV-mediated VIGS [3]. VIGS experiments often utilize bulbs, leaves [33,38,39,40], seeds [31,41,42], stem segments [3], and cotyledon segments as plant materials. In this study, we chose cuttings from the current year’s growth and seedlings with cotyledons as plant materials. Among them, the advantage of cuttings compared with the plant materials described above is that it is convenient to utilize materials that will not require cultivation for a long time to perform the VIGS experiments. The advantage of seedlings is that they have large cotyledons, rapid growth, and produce a silent phenotype in a short period time.

It is difficult to determine if the gene is silenced in a TRV-VIGS system when the silenced target gene has no distinct phenotypic characteristics [3]. To solve this problem, this study analyzed *CsPOR1* using a qRT-PCR analysis and observed silent plant leaf phenotypic changes to predict the timing of target gene silencing. The results showed that the gene silencing time is approximately 40 d. This study will greatly facilitate research on gene functions that have no phenotypic traits.

*CsTCS1* (caffeine synthase) is the most critical enzyme in the synthesis of caffeine. This study used the TRV-mediated VIGS system to reduce the expression of *CsTCS1* in tea leaves. The results of VIGS silencing showed that *CsTCS1* controls the biosynthesis of caffeine. After *CsTCS1* was inhibited, the levels of expression of the key genes *CsSAM* and *CsTIDH* involved in caffeine synthesis were also reduced. Based on the results, the new leaves of the cuttings grew pale pink when *CsTCS1* was silenced, and we hypothesized that the silencing of *CsTCS1* in the leaves could have inhibited the genes related to the control chlorophyll synthesis [43] and upregulated the expression of genes that control anthocyanin synthesis [44], resulting in the changes in leaf color in the *CsTCS1*-silenced plants. In addition, the accumulation of caffeine in tea leaves was reduced. These results indicate that the TRV-mediated VIGS system is suitable for the functional verification of tea tree genes and that this system is stable and reliable.

## 4. Materials and Methods

### 4.1. Plant Material and Growing Conditions

The first type of explant material was the F1 hybrid seeds of ‘Fuding Dabaicha’ (♀) × Longjing 43’ (♂) [45]. Seeds with the episperm removed were placed in a 4 cm tall tray that contained the nutrient solution for cultivation. The composition of the nutrients included 100 uL·L^−1^ of 6-benzylaminopurine (6-BA), 600 uL·L^−1^ of gibberellic acid (GA3), and 5 mL·L^−1^ of chloramphenicol. The seedlings were grown for 20 d at 25 °C in a greenhouse at Guizhou University (26°44′ N, 106°65′ E), Guiyang, China, with a relative humidity of 60–70% and 16 h/8 h light/dark. The seedlings were used for vacuum infiltration experiments when they had grown to 2–3 cm. The second type of explant material utilized was cuttings of Fuding Dabaicha grown in the current year [46], which were obtained from the Guizhou Academy of Agricultural Sciences (26°12′ N, 106°08′ E), Guiyang, China. Cuttings of the same growth status were selected for the vacuum infiltration experiments.

### 4.2. Total RNA Extraction

The total RNA was extracted using a TRIzol Up Plus RNA Kit (TianGen Biochemical Technology Co., Ltd., Beijing, China), following the manufacturer’s instructions. The RNA quality was checked by electrophoresis on a 1.0% (*w*/*v*) agarose gel. The cDNA was synthesized by reverse transcription using a Prime Script^TM^ II 1st strand cDNA Synthesis Kit (Beijing Solarbio Technology Co., Ltd., Beijing, China).

### 4.3. Cloning of a Partial Sequence of CsPOR1

Parts of the sequences of *CsPOR1* (GenBank MH925309.1) and *CsTCS1* (GenBank AB031280.1) were amplified using PrimeSTAR^®^ Max (Junotec Biotechnology Co., Ltd., Wuhan, China) high-fidelity DNA polymerase amplification of the target fragment. The PCR reaction system was 25 µL of PrimeSTAR Max Premix positive. A volume of 2.5 µL of the forward primer and reverse primer and 3.0 µL of cDNA were brought to a volume of 50 µL with sterile distilled water. The PCR reaction conditions were as follows: 95 °C for 5 min; 34 cycles of 95 °C for 30 s, 55 °C for 30 s, 72 °C for 1 min; 72 °C for 10 min, and storage at 4 °C. The PCR product was ligated into the cloning vector pMD18-T (Junuod Biotechnology Co., Ltd., Wuhan, China) and transformed into *E. coli* DH5α competent cells (Kewen Biotechnology Co., Ltd., Changsha, China) by heat shock. The cells were then plated overnight at 37 °C and cultured. Resistance to 50 mg/L of kanamycin and the universal primer M13-47/48 were used to amplify the bacterial liquid by PCR, and the positive recombinants that were screened were sent to the company for sequencing (Figure S3). The sequences were compared with those obtained from GenBank for confirmation (Table 3).

### 4.4. Vector Construction

**Figure 6:**
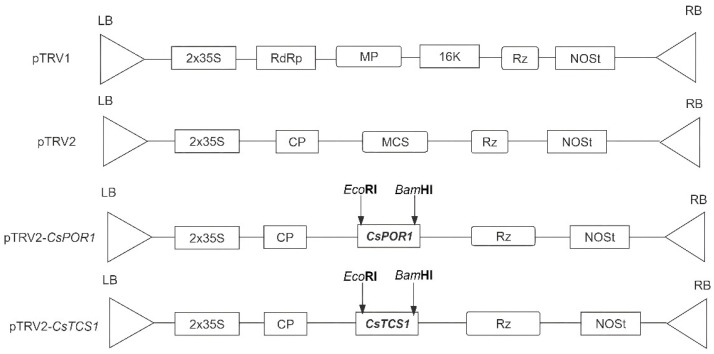
Map of the pTRV1, pTRV2, pTRV2-*CsPOR1,* and pTRV2-*CsTCS1* vectors used in this study. LB, left border; RB, right border; RdRp, RNA-dependent RNA polymerase; MP, movement protein; 16k, 16 kD protein; Rz, self-cleaving ribozyme; NOSt, NOS terminator; CP, coat protein; MCS, multiple cloning site.

The fragments of *CsPOR1* and *CsTCS1* used for VIGS were assembled into the pTRV2 virus vector (Figure 6).

Construction of the pTRV2-*CsPOR1* and pTRV2-*CsTCS1* vector. The pTRV2-*CsPOR1* vector was constructed using a 308 bp fragment of *CsPOR1* with *Eco*RI and *Bam*HI restriction sites. The PCR product and pTRV2 vector, which was digested by *Eco*RI and *Bam*HI restriction enzymes and recovered, were ligated using T4 DNA ligase (Covent Biotechnology Co., Ltd., Changsha, China) overnight at 16 °C, and then transformed into *E. coli* DH5α. The specific primers for pTRV2-*CsPOR1*-F and pTRV2-*CsPOR1*-R were used to verify the insertion of the 308 bp product by PCR with a bacterial solution (Table 3). Moreover, the 332 bp *CsTCS1* fragment was selected and cloned into the pTRV2 vector with *Eco*RI and *Bam*HI restriction sites using T4 DNA ligase (Covent Biotechnology Co., Ltd., Changsha, China).

### 4.5. Impact and Optimization of the Conversion Systems

#### 4.5.1. Tea Plant Infection

pTRV1, pTRV2, pTRV2-*CsPOR1*, and pTRV2-*CsTCS1* were introduced into the *Agrobacterium* GV3101 competent state using the freeze–thaw method. Each strain of *Agrobacterium* was inoculated into solid YEP media, which contained 100 mg of L^−1^ kanamycin and 50 mg·L^−1^ of rifampin, and the 28 °C activated culture was grown for 48 h. Single colonies were cultured in the corresponding liquid YEP media until the cells reached an OD_600_ of 1.0–1.5. The *Agrobacterium* cells were centrifuged at 6000 rpm for 6 min. The bacterial fluid was collected and suspended in 4.74 g·L^−1^ of MS, 2 mol·L^−1^ of 6-BA, 2 mol·L^−1^ of acetosyringone (AS), 100 umol·L^−1^ of 1-napthalene acetic acid (NAA), and 16 g·L^−1^ of MgCl_2_, pH 5.6. The OD_600_ was adjusted to 1.2. pTRV1 was mixed with pTRV2, pTRV2-*CsPOR1*, and pTRV2-*CsTCS1* bacterial liquid 1:1, and the two bacterial liquids were fully mixed at room temperature [47,48]. The two types of tea plant materials were vacuum infiltrated. This process was conducted on the tea seedlings using a blade to introduce 3–4 wounds around the cotyledons. All the leaves were removed, and the tea seedlings were placed in a Buchner flask that contained mixed bacterial liquid and vacuum infiltrated. The inoculated seedlings were grown in a greenhouse at 25 °C at 16 h/8 h light/dark. Shears were used to cut the tea cuttings to 15–20 cm. A slice of a mature leaf was retained, and the tea cuttings were then placed in a Buchner flask that contained mixed bacterial liquid for vacuum infiltration. They were kept in the dark for three days and then grown at 25 °C in a greenhouse with a 16 h/8 h light/dark cycle.

#### 4.5.2. Optimization of the Transformation System

Two-factor multi-level experiments identified the most suitable vacuum pressure and OD_600_ of *Agrobacterium* for the TRV-mediated VIGS system of tea. The same conditions were used for the vacuum infiltration time, osmosis buffer components, and soaking time. *Agrobacterium* strains that contained pTRV1, pTRV2, and pTRV-*CsPOR1* were resuspended in the two different infiltration buffers, and the final OD_600_ values of 1, 1.2, and 1.5 were obtained, respectively. The vacuum pressure (0.6 kPa, 0.7 kPa, and 0.8 kPa) and OD_600_ (1, 1.2, and 1.5) were combined in pairs and divided into nine groups to explore the best conditions. *Agrobacterium* pTRV1 and pTRV2, and pTRV1 and pTRV-*CsPOR1*, were mixed at a 1:1 ratio and incubated for 1 or 2 h at room temperature. Multiple tea seedlings and cuttings were randomly selected and infiltrated with different vacuum pressures and optical densities of *Agrobacterium.*

### 4.6. Silencing and HPLC Analysis of CsTCS1 for Caffeine Content

The *CsTCS1* involved in caffeine biosynthesis was silenced in the tea plant using the optimal system that resulted from Section 2.5, and a non-silenced plant was used as the control. The non-silenced plants were infected with pTRV1 + pTRV2 *Agrobacterium*. When the *CsTCS1* gene was silenced, the caffeine in the leaves of the *CsTCS1*-silenced plants, wild-type plants, and non-silenced plants was determined by high-performance liquid chromatography (HPLC) (Hitachi Limited Co., Ltd., Tokyo, Japan). A total of 1.0 g of ground tea sample was weighed on a scale that was accurate to 0.0001 g and placed in a 500 mL flask. A total of 4.5 g of magnesium oxide and 300 mL of ultrapure water were brought to the boil in a boiling water bath and extracted for 20 min. The flask was shaken every 5 min. After extraction, the liquid was immediately filtered under heat and reduced pressure. The filtrate was transferred to a 500 mL volumetric flask, and after it had cooled, the volume was brought up to scale with ultrapure water and mixed well. A portion of the test solution was passed through a 45 PM membrane filter [49]. The conditions for the HPLC included a detection wavelength of 240 nm; a mobile phase of methanol: ultrapure water (3:7, *v/v*); a flow rate of 1 mL/min; a column temperature of 40 °C; and an injection volume of 10 ul, followed by ISO 17027:1995 (2013) (ISO). The relative content of caffeine was calculated using Equation (1).

(1) $$\mathrm{Caffeine}\;\left(\%\right)=\frac{C_{1}\times V_{1}}{m\times \omega \times 10^{6}}\times 100\%$$

### 4.7. Analysis of Expression by qRT-PCR

The total RNA was extracted using a TRIzol-A+ kit (TianGen Biochemical Technology Co., Ltd., Beijing, China). The cDNA was synthesized by reverse transcription using a PrimeScript^TM^ II first strand cDNA Synthesis Kit (Solarbio Technology Co., Ltd., Beijing, China) and diluted 10-fold for real-time PCR detection. To determine the levels of expression of the *CsPOR1* and *CsTCS1* genes, Primer Premier 5.0 was used to design specific primers for *CsPOR1* and *CsTCS1*. The actin gene from tea was used as an internal reference (Table 1). A real-time quantitative reverse transcription PCR (qRT-PCR) assay was performed on a Bio-Rad CFX Connect^TM^ real-time quantitative PCR instrument (Bio-Rad, Hercules, CA, USA). The qRT-PCR used was a general-purpose high-sensitivity dye-based quantitative PCR detection kit from Nanjing Novozan Biotechnology Co., Ltd. (Nanjing, China). The qRT-PCR reaction system was utilized according to the manufacturer’s instructions. The conditions and system of the qRT-PCR were as follows: one cycle of denaturation (94 °C, 5 min), followed by 40 cycles of amplification (94 °C, 30 s; 50 °C, 45 s) and signal acquisition (72 °C, 113 s); 50 µL of the qRT-PCR selection system, which included 20 µL of nuclease-free water, 25 µL of Pfu PCR Mix (Biorun Biotechnology Co., Ltd., Wuhan, China), 1 µL of template, 2 µL of primer(+) (100 μM), and 2 µL of primer(-) (100 μM). The relative levels of expression of the genes were analyzed using 2^−ΔCt^.

### 4.8. Data Analysis

The data were subjected to an analysis of variance (ANOVA) using SPSS 18.0 (IBM, Inc., Armonk, NY, USA); error bars represent the mean ± standard deviation of data from the three independent experiments. Different letters indicate significant differences at *p* < 0.05 and extremely significant differences at *p* < 0.01. The qRT-PCR data were analyzed using SPSS for the significant difference analysis (*p* < 0.05). The data were analyzed using Microsoft Excel 2007 (Redmond, WA, USA), and a sequence analysis was performed using DNAMAN 5.0 and the database from a public website (https://www.ncbi.nlm.nih.gov/, accessed on 8 June 2022).

## 5. Conclusions

This study attempted to ameliorate the lack of a complete genetic transformation system for tea plants and other problems. Tea was genetically transformed without the need for tissue culture for the first time by utilizing a TRV-VIGS system. This method provides an effective method to study the function of genes in tea plants and accelerate the process of functional genome research in tea.

## Figures and Tables

**Table 1:** The efficiency of infection in different vacuum pressures and *Agrobacterium* densities (Tea seedlings).

Combinations of Infiltration Buffer	OD_600_	Vacuum Pressure	Number of Infected Seedlings	Number of Successful Infected Seedlings	Infection Efficiency (%)
4.74 g·L^−1^ MS + 2 mol·L^−1^ 6-benzylaminopurine + 2 mol·L^−1^ acetosyringone + 100 umol·L^−1^ 1-naphthylacetic acid	1.0	0.6	40	0	0
	1.2	0.6	40	0	0
	1.5	0.6	40	0	0
	1.0	0.7	40	0	0
	1.2	0.7	40	5	12.5
	1.5	0.7	40	0	0
	1.0	0.8	40	0	0
	1.2	0.8	40	1	2.5
	1.5	0.8	40	0	0

**Table 2:** The efficiency of infection following different vacuum pressures and *Agrobacterium* densities (Tea cuttings).

Combinations of Infiltration Buffer	OD_600_	Vacuum Pressure	Number of Infected Cuttings	Number of Successful Infected Cuttings	Infection Efficiency (%)
4.74 g·L^−1^ MS + 2 mol·L^−1^ 6-benzylaminopurine + 2 mol·L^−1^ acetosyringone + 100 umol·L^−1^ 1-naphthylacetic acid + 16 g·L^−1^ MgCl_2_	1.0	0.6	25	0	0
	1.2	0.6	24	0	0
	1.5	0.6	20	0	0
	1.0	0.7	25	0	0
	1.2	0.7	25	0	0
	1.5	0.7	26	0	0
	1.0	0.8	24	1	4.16
	1.2	0.8	23	8	34.7
	1.5	0.8	26	2	7.69

**Table 3:** List of primers used in this study.

Usage	Primer Name	Primer Sequence
Partial gene cloning	*CsPOR1*	F: CCAAGGCTCTAGCTGAAACA R: GAAAGGAGGAAGTGTCCAAGAT
Partial gene cloning	*CsTCS1*	F: CTGAATTGGTTTCACAGGGATTG R: TGTGGTCTTCGGTAGCTTTG
TRV virus testing	pTRV1	F: AAAGTGACTGGTGTGCCTAAA R: CTTGCAGAGCAGGAACTCTATC
TRV virus testing	pTRV2	F: CTACTACTACCAAGGCGAACAC R: GTCGTCAAGCCACTTCCTAA
Vector construction	pTRV2-*CsPOR1*	F: GAGGGTGGGTGATACCATATTC R: GCTGCTGTTTGTGCTCTTATAG
Vector construction	pTRV2-*CsTCS1*	F: GACACCTTCAATATACCCAGCTA R: ACTAGGATGATACTTGTGGTCTTC
qRT -PCR	qRT -PCR *CsPOR1*	F: CCAAGGCTCTAGCTGAAACA R: GTCAAGTGAGGCAAGGTCTAA
qRT -PCR	qRT -PCR *CsTCS1*	F: ACAAGCCCTCCTGTTGTAAG R: CCTCTTGGGATCTAGCATTGAG
qRT-PCR	qRT -PCR *CsTIDH*	F: GCGCCATCCGACTATGATTT R: CCACTACTTGTCCTCCATCTTTC
qRT-PCR	qRT -PCR *CsSAM*	F: CGATGAGACCGTGACAAATGA R: GAAGGGTTGAGGTGGAAGATG
qRT-PCR	Actin	F: CAGACCGTATGAGCAAGGAAAT R: GTGCTTAGGGATGCAAGGATAG

## Data Availability

Not applicable.

## Supplementary Materials

The following supporting information can be downloaded at: https://www.mdpi.com/article/10.3390/ijms24010392/s1.
