# Supplementary material for: Establishment of a Virus-Induced Gene-Silencing (VIGS) System in Tea Plant and Its Use in the Functional Analysis of CsTCS1
Source: Int J Mol Sci. 2022 Dec 26;24(1):392. doi: 10.3390/ijms24010392 (PMC9820744; doi:10.3390/ijms24010392)
Supplement: Supplementary file 1 [file ijms-24-00392-s001.zip › ijms-2090326-supplementary.pdf]

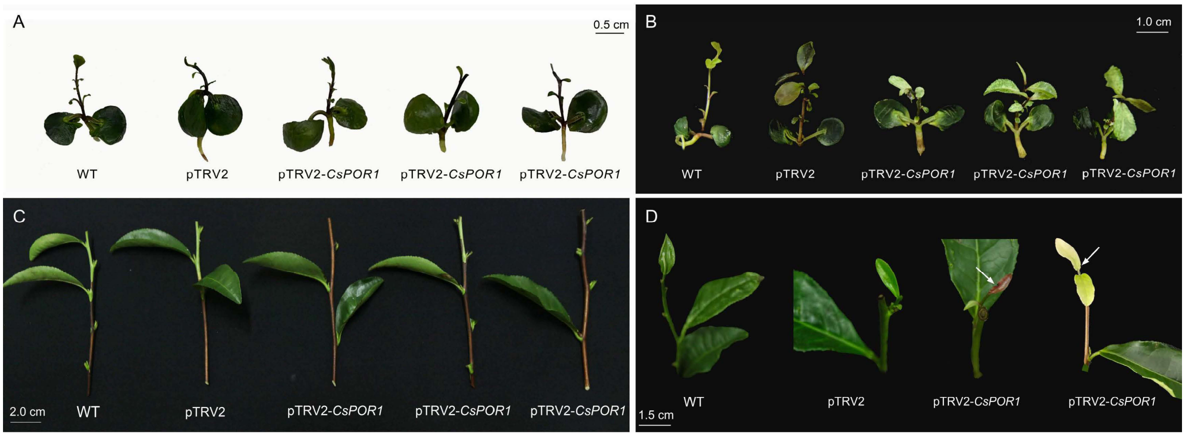

Figure S1. Construction and validation of the recombinant viral vector pTRV2-*CsPOR1* and pTRV2-*CsTCS1*. (A,C) Construction and validation of the recombinant viral vector pTRV2-*CsPOR1*. (B,D) Construction and validation of the recombinant viral vector pTRV2-*CsTCS1*.

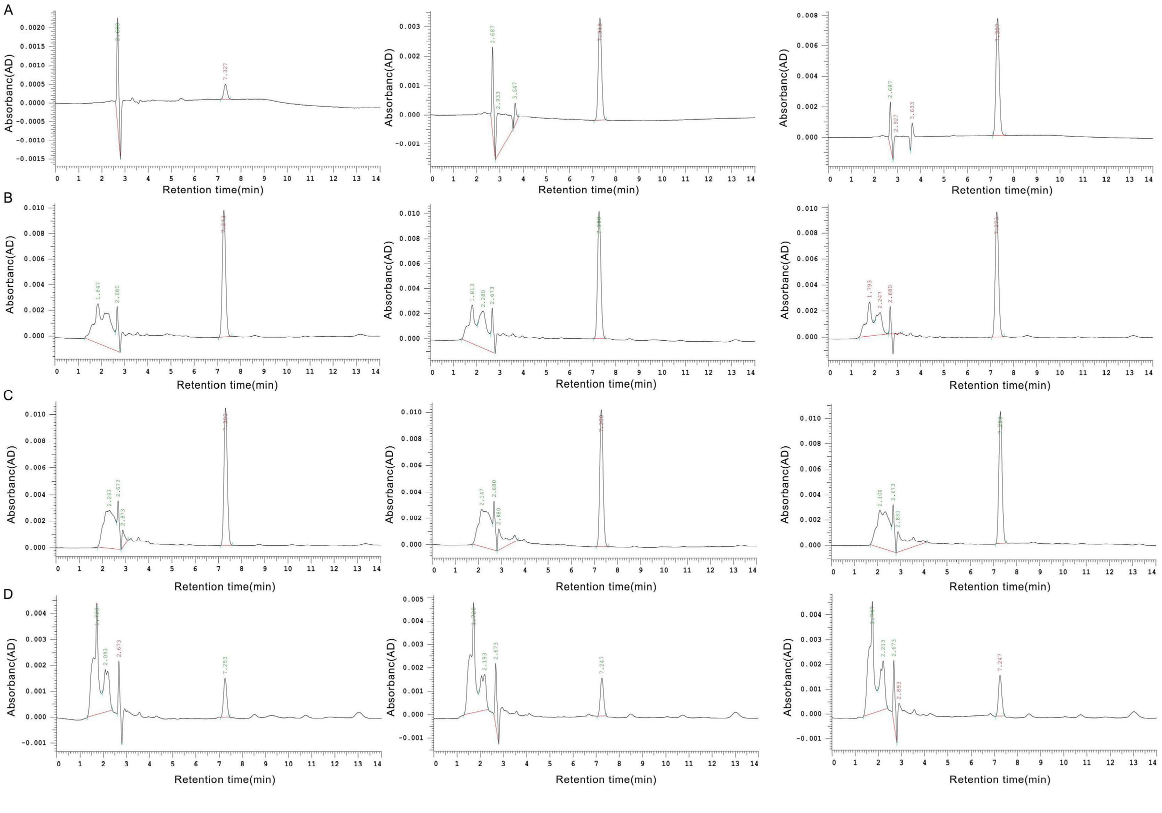

Figure S2. HPLC analysis of caffeine content in the leaves of *CsTCS1* silent plants. (A) Peak diagram of caffeine standard (10 ug·mL^{-1}, 50 ug·mL^{-1}, 100 ug·mL^{-1}); (B) untreated tea plants; (C) infection with pTRV1 + pTRV2; (D) infection with pTRV1 + pTRV2-*CsTCS1*. HPLC, high-performance liquid chromatography.

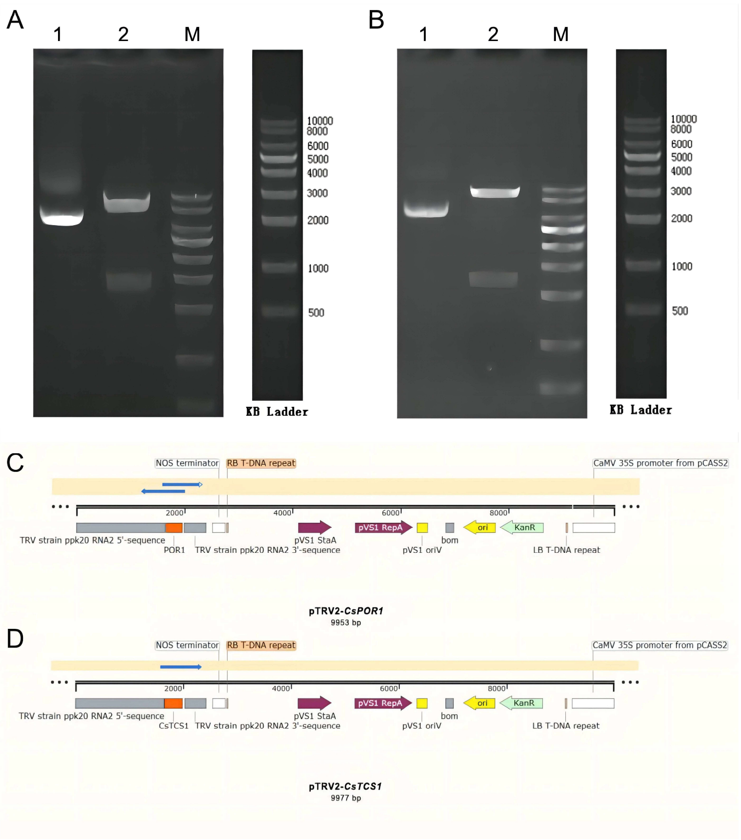

Figure S3. Comparison of tea plants before and after infection with pTRV1 + pTRV2-*CsPOR1 Agrobacterium*. (A,B) Image of tea plants before the pTRV1 + pTRV2-*CsPOR1 Agrobacterium* infection. (C,D) Image of tea plants after the pTRV1 + pTRV2-*CsPOR1 Agrobacterium* infection.

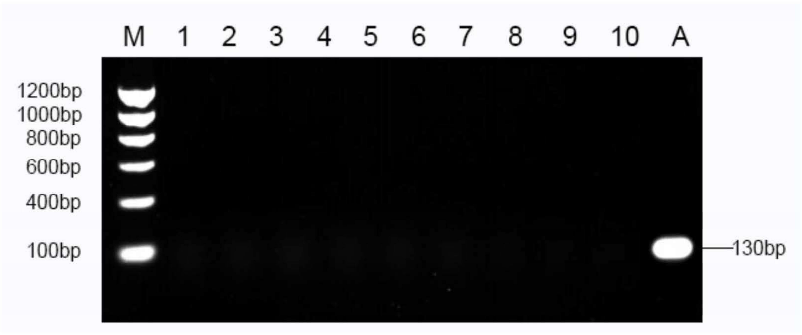

Figure S4. Detection of Agrobacterium contamination. 1, uninfected seedlings; 6, uninfected cuttings; 2 and 7, seedlings infected with pTRV1 + pTRV2; 3 and 8, cuttings infected with pTRV1 + pTRV2; 4, seedlings infected with pTRV1 + pTRV2-*CsPOR1*; 5, cuttings infected with pTRV1 + pTRV2-*CsPOR1;* 9, seedlings infected with pTRV1 + pTRV2-*CsTCS1*; 10, cuttings infected with pTRV1 + pTRV2-*CsTCS1.* A, positive control. M: DL1200 marker.
